# Differential Upregulation and Functional Activity of S1PR1 in Human Peripheral Blood Basophils of Atopic Patients

**DOI:** 10.3390/ijms232416117

**Published:** 2022-12-17

**Authors:** Natalie Gray, Maren M. Limberg, Daniela Wiebe, Tobias Weihrauch, Anna Langner, Nicola Brandt, Anja U. Bräuer, Ulrike Raap

**Affiliations:** 1 Division of Experimental Allergy and Immunodermatology, School of Medicine and Health Sciences, Carl von Ossietzky University Oldenburg, 26129 Oldenburg, Germany; 2 Division of Anatomy, School of Medicine and Health Sciences, Carl von Ossietzky University Oldenburg, 26129 Oldenburg, Germany; 3 Research Center for Neurosensory Science, Carl von Ossietzky University Oldenburg, 26129 Oldenburg, Germany; 4 University Clinic of Dermatology and Allergy, University of Oldenburg, 26133 Oldenburg, Germany

**Keywords:** basophils, S1P, S1PR, atopy, apoptosis, chemotaxis

## Abstract

Basophils are key effector cells in atopic diseases, and the signaling sphingolipid Sphigosine-1-phosphate (S1P) is emerging as an important mediator in these conditions. The possible interaction of S1P and basophils and the resulting biological effects have not yet been studied. We hypothesize that S1P influences the function of basophils in atopy and aim to elucidate the modes of interaction. S1P receptor (S1PR) expression in human peripheral blood basophils from atopic and non-atopic patients was assessed through qRT-PCR and flow cytometry analysis. Functional effects of S1P were assessed through a basophil activation test (BAT), calcium flux, apoptosis, and chemotaxis assays. Immunofluorescence staining was performed to visualize intracellular S1P. Human basophils express S1PR1, S1PR2, S1PR3, and S1PR4 on the mRNA level. 0.1 µM S1P have anti-apoptotic, while 10 µM exhibits apoptotic effects on basophils. Basophils from atopic patients show less chemotactic activity in response to S1P than those from healthy donors. Protein expression of S1PR1 is downregulated in atopic patients, and basophils in lesional AD skin possess intracellular S1P. These findings suggest that the interaction of S1P and basophils might be an important factor in the pathophysiology of atopy.

## 1. Introduction

Though basophil granulocytes are the least abundant type of leukocyte in the human body, making up less than 1% of circulating white blood cells, they are a pivotal player in a variety of human diseases, including atopic diseases [1,2,3]. They notably contribute to the pathophysiological process in hypersensitivity reactions (e.g., allergic rhinitis [4], anaphylaxis [5]), asthma [6], contact dermatitis [7], and atopic dermatitis (AD) [8]. Classically being activated by crosslinking of IgE-antibodies bound to the high-affinity IgE-receptor (FcεRIα) [9,10], basophils release pro-inflammatory mediators such as histamine [11] and leukotriene (LTC4) [12]. Additionally, they are a major source of the immunomodulation T_h_2 type cytokines interleukin 4 (IL-4) [13] and IL-13 [14], as well as IL-31, which is a key player in mediating the sensation of itch [15], and are therefore a major initiator of allergic inflammation [16]. Basophil activation, function, proliferation, and lifespan are critically influenced by IL-3, a cytokine released mainly from activated T-cells [1,17,18]. In various cases, IL-3 priming relevantly enables and enhances basophil function in response to stimuli [19,20].

Though basophils are most known for being activated through IgE-dependant pathways, alternative activation pathways through different channels, such as IgG [21] and IgD-antibodies [22], have been observed, resulting in different basophil functions. Antibody-unrelated pathways of activation include cytokines like IL-3 and thymic stromal lymphopoietin (TSLP) [23], proteases [24], and pathogen-associated stimuli like glycoproteins [25,26]. Although basophils most often exhibit pro-inflammatory properties, evidence is emerging that, in some cases, they might also be capable of inducing anti-inflammatory effects, such as releasing interleukin 10 (IL-10) [27,28,29,30].

The pleiotropic, bioactive lipid, and second messenger Sphingosine-1-phosphate (S1P) is a key regulator of immune responses in health and disease [31,32]. Thus far, five G-protein coupled, cell surface S1P receptors (S1PRs) have been discovered and are expressed widely with inter-cell type and organ variability [33]. The receptors bind S1P with a high affinity and mediate a variety of biological functions, including cell proliferation [34], angiogenesis [35], apoptosis [36], and cell migration [37,38]. S1PR1, in particular, is responsible for lymphocyte egress from the secondary lymphoid organs such as the lymph nodes and thymus [38,39]. S1PR1 mediates cell migration in various types of cells, such as B-cell displacement to splenic follicles [40], NK T-cell emergence into peripheral tissues [41], and chemo- and fugetaxis in T-ALL blasts [42]. Thus, in this paper, we further investigate the role of the signaling sphingolipid S1P on the activation and function of human basophils.

The S1P/S1PR axis has been found to be dysregulated in various atopic conditions, such as asthma, anaphylaxis, food allergies, and atopic dermatitis, all of which also have high levels of basophil involvement [33,43]. In asthmatics, S1P levels significantly increase in bronchoalveolar lavage fluid (BALF) after antigen exposure [44,45], and the local application of S1P in an asthma mouse model exacerbates symptoms [46]. In healthy donors, S1P serum concentrations range from 0.4 to 1.1 µM [47,48,49], and S1P concentrations in serum from AD patients have been observed to be elevated and correlated with disease severity [50]. The application of S1PR modulators has shown promising results in reducing AD symptoms in both animal studies and human clinical trials [32,51].

S1PR expression has been investigated for various immune cells, including eosinophils [52], neutrophils [53], and mast cells [54,55], but until now, this has not been the case for basophils. The results presented in this research article, therefore, reveal a completely novel perspective on possible basophil functions mediated through the newly discovered S1P receptors.

As both basophils and S1P have important roles in the pathophysiology of atopic conditions, we were intrigued to study both in combination and potentially uncover an important link to further understand disease progression.

## 2. Results

### 2.1. Basophils Express S1PR1, S1PR2, S1PR3, and S1PR4 on the mRNA Level

**Figure 1:**
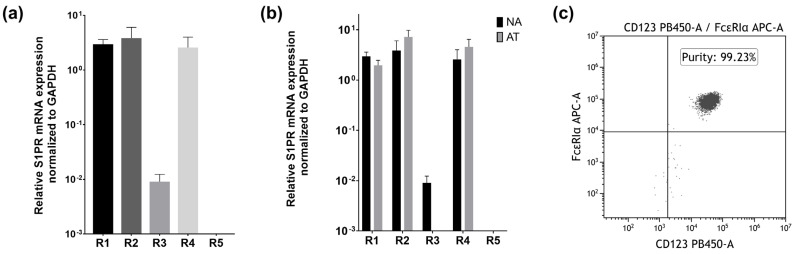
mRNA S1PR expression in human basophils of NA and AT patients. NA = non-atopic, AT = atopic. R1 = S1PR1, R2 = S1PR2, R3 = S1PR3, R4 = S1PR4, R5 = S1PR5. (**a**) qPCR analysis of S1PR mRNA expressions. Basophils express S1PR1, S1PR2, S1PR3 and S1PR4 on an mRNA level (*n* = 4). (**b**) Comparison of mRNA S1PR expression in NA and AT patients (*n* = 4). (**c**) Representative dot plot demonstrating basophil purity determined through selecting anti-CD123 PB and anti-FcεRIα APC stained cells. Data are shown as mean + SEM.

As data regarding S1PR expression exist for most immune cells but not yet basophils, we wanted to investigate if they also express these receptors. mRNA expression levels of all five S1P receptors were analyzed through quantitative real-time PCR, and measured expression levels were normalized to the housekeeping gene GAPDH. Human basophils of non-atopic donors expressed S1PR1, S1PR2, and S1PR4 in comparable high levels on the mRNA level, while receptor 3 was expressed at much lower rates (Figure 1a). Expression of S1PR5 was also investigated, but as no signal was detectable, this suggests that basophils express very low to no amounts of the mRNA transcript. mRNA expression in basophils from atopic patients is comparable to that in non-atopic patients. While slight decreases in S1PR1 and S1PR3 expression and small increases in S1PR2 and S1PR4 expression could be detected, this is not enough to be statistically significant. As basophil purity after isolation was consistently ≥96% (Figure 1c), these results can be confidently attributed to basophils themselves and are unlikely to stem from contaminating cells.

### 2.2. S1P Does Not Influence Basophil CD63 or CD203c Expression in Basophils from NA Patients

**Figure 2:**
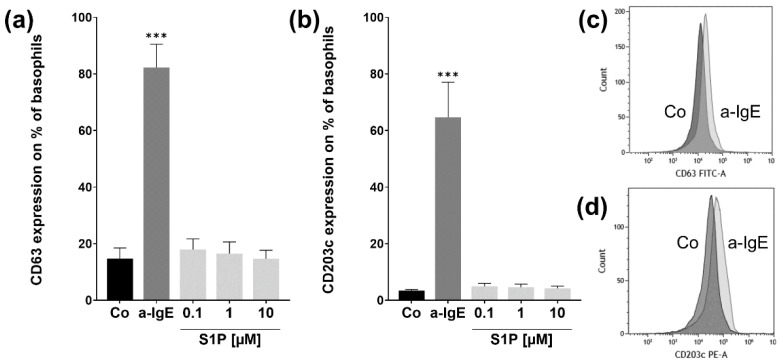
Basophil activation test assay. Flow cytometry analysis of CD63 & CD203c protein expressions after stimulation with S1P. Basophils were stimulated with fatty acid free BSA (4 mg/mL) (Co), S1P (0.1 µM, 1 µM, 10 µM) or a-IgE (1 µg/mL) for 30 min (*n* = 5). (**a**) CD63 protein expression is significantly increased for the positive control a-IgE. (**b**) CD203c expression is markedly higher in a-IgE than in Co-stimulated cells. (**c**) Representative overlay histogram of CD63 expression in Co (dark grey) and a-IgE stimulated (light grey) basophils. (**d**) Representative overlay histogram of CD203c expression in Co (dark grey) and a-IgE stimulated (light grey) basophils. Data are shown as mean + SEM, *p*-values (*** ≤ 0.001). Significances are calculated in relation to the Co group.

Since atopy is mostly associated with IgE-mediated degranulation of basophils, we investigated if the stimulation of S1P receptors with different concentrations of S1P had an effect on the basophil degranulation assessing markers CD63 and CD203c, which are markers for anaphylactic and piecemeal degranulation, respectively. The number of CD63-positive basophils significantly increased after stimulation with the positive control a-IgE (*p* < 0.001; a-IgE: 82.2% ± 8.26; Co: 14.7% ± 3.78) (Figure 2a). A similar increase could be observed in the number of CD203c-positive basophils after stimulation with a-IgE (*p* < 0.001; a-IgE: 64.6% ± 12.45; Co: 3.4 & ± 0.43) (Figure 2b). The shift in marker expression is further visualized in Figure 2c,d. The three different doses of S1P had no significant effect on CD63 or CD203c expression. Thus, S1P-inducing degranulation in basophils is unlikely.

### 2.3. S1P Does Not Affect Short Term Cytosolic Ca^2+^ Levels in Basophils from NA Patients

**Figure 3:**
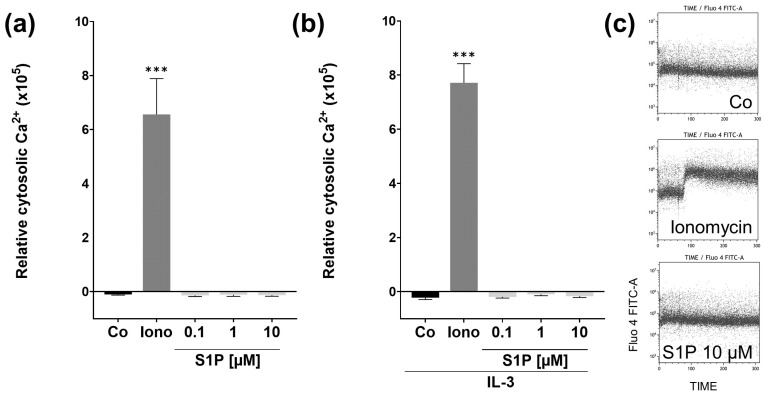
Calcium flux assay. Changes in basophil cytosolic Ca ^2+^ levels after stimulation with S1P. Purified basophils were incubated with Fluo-4 AM (4 µM), and calcium flux was measured by flow cytometry. (**a**) Differences in relative cytosolic Ca^2+^ concentrations to baseline, after stimulation with 500 nM ionomycin (positive control), 0.1, 1, or 10 µM S1P (*n* = 6). (**b**) Changes in relative cytosolic Ca^2+^ concentrations after priming with IL-3 (10 ng/mL) and stimulation with either ionomycin or different concentrations of S1P (*n* = 4). (**c**) Changes in fluorescence over 300 s during application of RPMI, ionomycin, and 10 µM S1P. Data are shown as mean + SEM, *p*-values (*** ≤ 0.001). Significances are calculated in relation to the Co group.

To determine whether the binding of S1P to S1PRs causes the activation of pathways associated with a change in cytosolic Ca^2+^ levels, we next performed calcium-flux assays. Fluo-4 loaded basophils were placed in the flow cytometer, and stimuli were added while measuring the intracellular Ca^2+^ level-associated fluorescence in real-time (Figure 3a). As we could observe no effects on Ca^2 +^ -flux and IL-3 is a potent priming agent for basophils, often enabling or enhancing responses to other stimuli, we repeated the experiment with basophils preincubated with 10 ng/mL IL-3, in case S1P/S1PR signaling is also IL-3 dependant (Figure 3b). For both assays, the positive control ionomycin was able to significantly (*p* < 0.001) increase cytosolic Ca^2+^ concentrations, but the different concentrations of S1P did not influence calcium flux in any significant ways during the 300 s of observation. This does not exclude the possibility of reactions occurring outside of the observed time frame.

### 2.4. S1P Has Pro- and Antiapoptotic Effects on Basophils from NA Patients

**Figure 4:**
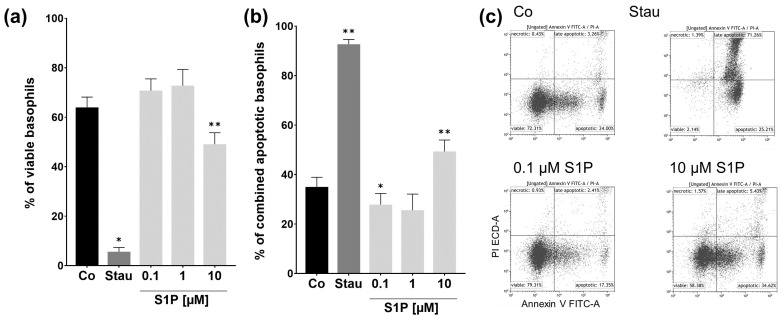
Apoptosis assay. Percentage of viable and combined apoptotic and late apoptotic basophils after S1P stimulation. Flow cytometry analysis of the annexin V-FITC and PI apoptosis assay. Basophils were stimulated with fatty acid free BSA (Co), S1P (0.1 µM, 1 µM, 10 µM), or staurosporine (1 µM) for 24 h (*n* = 3). (**a**) Percentage of viable basophils. (**b**) Percentage of combined apoptotic and late apoptotic basophils. Stimulation with 10 µM S1P decreases the number of viable and increases the number of apoptotic cells. 0.1 µM S1P reduces the number of apoptotic basophils. (**c**) Representative dot plots demonstrate the different stages of apoptosis for Co, Ionomycin, and 10 µM S1P stimulated basophils. Data shown as mean + SEM, *p*-values (* ≤ 0.05; ** ≤ 0.01). Significances are calculated in relation to the Co group.

As the preceding experiments suggest that stimulation with S1P does not cause an immediate effect or reaction in basophils, we wanted to investigate what effect might be observable after longer incubation periods. Due to the fact that basophils have a relatively short lifespan of about 1 to 2 days in the peripheral blood, the possibility of S1P influencing this lifespan and, therefore, apoptosis came to mind. Hence, we performed an annexin V-FITC and PI apoptosis assay after incubation with stimuli for 24 h. Annexin V-FITC was used to stain the apoptosis marker phosphatidylserine, and PI to stain nucleic acids to together determine the stages of apoptosis. Percentages of viable cells (Figure 4a) and combined apoptotic cells (apoptotic + late apoptotic) (Figure 4b) were analyzed individually. The numbers of necrotic cells were not analyzed, as they are only measurable for a very brief period of time before losing all markers assessed in the assay. The average percentage of viable cells in the apoptosis-inducing staurosporine (Stau) positive control group was significantly lower than the mean of the unstimulated control group (*p* = 0.0101, Stau.: 5.6% ± 1.73; Co: 64% ± 4.17). The number of viable 10 µM S1P treated basophils was also markedly decreased in comparison to the control group (*p* = 0.0012, 10 µM S1P: 49.1% ± 4.67; Co: 64% ± 4.17). This was also reflected in the combined apoptotic populations. The mean number of apoptotic cells dramatically increased for staurosporine treated basophils, and for 10 µM S1P treated cells (Co: 35% ± 3.89; Stau: *p* = 0.0098, 92.7% ± 1.89; 10 µM S1P: *p* = 0.0029, 49.3% ± 4.65). Interestingly, the percentage of apoptotic basophils decreased in basophils stimulated with 0.1 µM S1P (*p* = 0.0418, 0.1 µM S1P: 27.8% ± 4.48; Co: 35% ± 3.89). A similar trend was observable for 1 µM treated basophils, though this value is not statistically significant. This trend was also reflected in the slightly increased number of viable basophils for lower S1P concentrations (0.1 and 1 µM). These results suggest that high doses of S1P have an apoptotic effect on human basophils, while the low dose of 0.1 µM seems to possess anti-apoptotic properties.

### 2.5. S1PR1 Protein Expression and Basophil Chemotaxis Differ in NA and AT Patients

**Figure 5:**
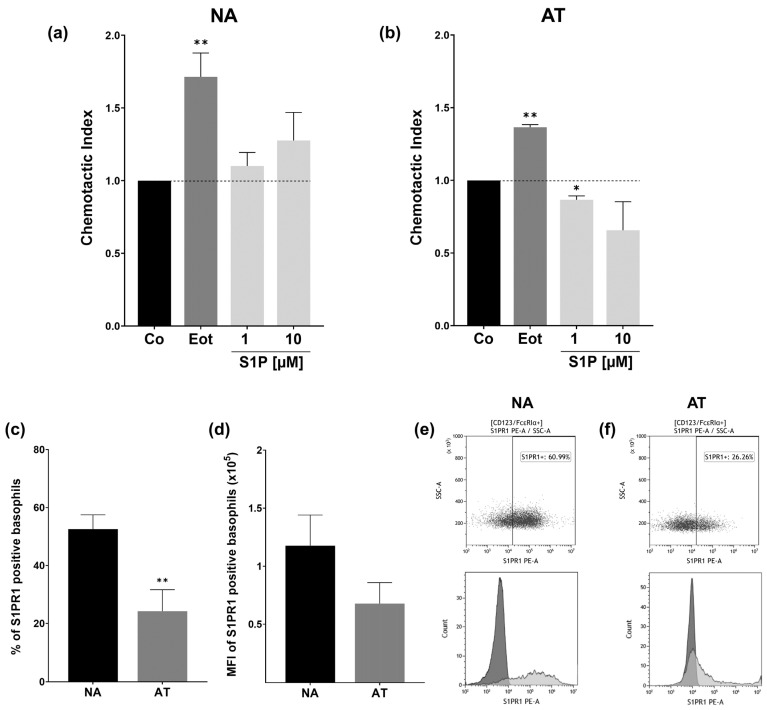
Chemotaxis assay and S1PR1 protein expression. Basophil chemotactic activity and flow cytometry analysis of S1PR1 protein expression in NA and AT patients. NA = non-atopic, AT = atopic, SSC = side scatter. Basophils were placed in the upper chamber of a transwell migration assay, and chemotactic activity toward stimuli in the bottom chamber was measured. Cells were counted after 3 h through flow cytometry. Fatty acid free BSA (Co), S1P (1 and 10 µM), or eotaxin (Eot) (8 ng/mL) were applied in FCS-free RPMI. The chemotactic index was calculated in relation to the transmigrated cells in the Co group. (**a**) Chemotactic index of NA basophils (*n* = 6). Basophils are significantly attracted to eotaxin. (**b**) Chemotactic index of AT basophils (*n* = 3). Basophils migrate towards eotaxin, whereas 1 µM S1P significantly reduces the number of transmigrating cells. (**c**) Flow cytometry analysis of protein level S1PR1 expression. Percent of S1PR1 positive basophils of NA (*n* = 6) and AT (*n* = 5) patients. AT patients express significantly less S1PR1 on the cell surface than the NA group. (**d**) Flow cytometry analysis of S1PR1 MFI in NA (*n* = 6) and AT (*n* = 5) basophils. (**e**) Representative dot plot of S1PR1 protein expression in NA basophils (top) and representative histogram of S1PR1 (light grey) and isotype control (dark grey) stained NA basophils (bottom). (**f**) Representative dot plot of S1PR1 protein expression in AT basophils (**top**) and representative histogram of S1PR1 (light grey) and isotype control (dark grey) stained AT basophils (**bottom**). Data shown as mean + SEM, *p*-values (* ≤ 0.05; ** ≤ 0.01). Significances are calculated in relation to the Co or NA group.

On account of S1PR signaling often being involved in the migration and chemotaxis of immune cells, we also wanted to perform a chemotaxis assay to determine if this is also the case for basophils. Basophils were placed in the top chamber of a transwell migration assay set-up, and movement towards stimuli in the bottom chamber was determined through flow cytometry counting after 3 h. This assay was performed separately for both non-atopic (Figure 5a) and atopic (Figure 5b) donors. The chemotaxis index (CI) was calculated by normalizing cell numbers to the number of migrated cells in the control group, explaining why the value for Co is always 1.

For non-atopic donors, as expected, basophils clearly migrated towards the positive control eotaxin (*p* = 0.0072, CI = 1.72 ± 0.163). Although S1P seemed to also have a slightly dose-dependent chemotactic effect on basophils, this is not enough to be significant. We observed high variability between donors, with the effects of S1P on the basophils ranging from highly chemotactic to slightly chemorepulsive. As we observed reduced chemotactic in basophils from an atopic donor, we decided to further investigate the effects in the atopic group. The effect of eotaxin on basophils of atopic patients was comparable to that of non-atopic donors, with cells significantly migrating towards eotaxin (*p* = 0.026, CI = 1.37 ± 0.019). S1P, on the other hand, seemed to have the opposite effect in comparison to non-atopic donors. While 1 µM had a clear (*p* = 0.0380) fugetactic effect on basophils (*p* = 0.0380, CI = 0.87 ± 0.27), and the effect visually seemed to be even more pronounced for 10 µM, high inter-patient variabilities prevent this statistic from being significant. As S1PR1 has been found to strongly mediate chemotaxis and cell migration in other immune cells, we suspected that this might also be the case for basophils and that the reduced cell migration in atopic patients might be due to receptor expression variability on the protein level. We consequently investigated the protein level S1PR1 expression in both groups through anti-S1PR1 staining and flow cytometry analysis. We found that the number of S1PR1-expressing basophils was significantly reduced in atopic patients (*p* = 0.0096, NA: 52.6% ±4.95; AT: 24.2% ±7.44) (Figure 5c). A thorough analysis of S1PR1 mean fluorescence intensity (MFI) showed a similar downward trend for AT basophils. This was not statistically significant due to high variability (Figure 5d).

### 2.6. S1P Is Expressed Intracellularly in the Basophils of an AD Patient

**Figure 6:**
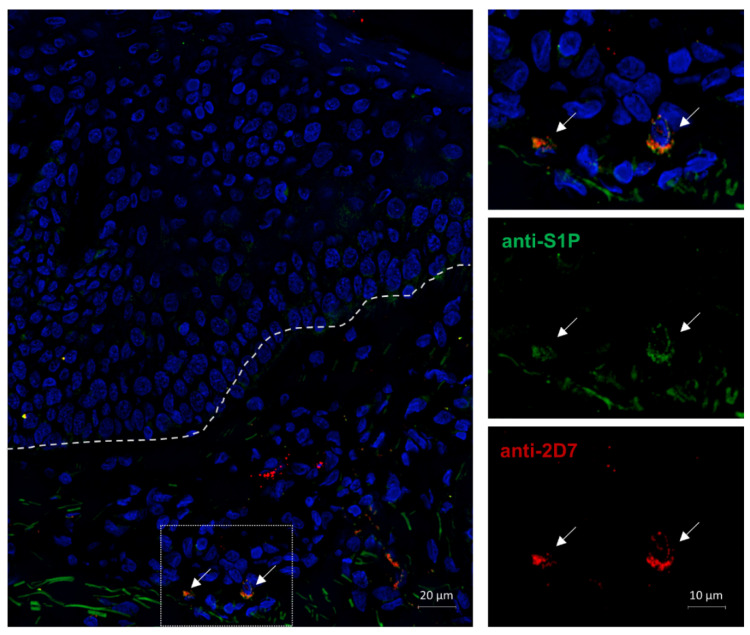
S1P immunofluorescence staining of S1P positive basophils in AD skin. AD = atopic dermatitis. A lesional AD skin biopsy was fixed in 4% PFA, and a double immunofluorescence staining was performed. Antibodies against the basophil marker 2D7 (red) and the lipid S1P (green) were utilized. Nuclei were stained with DAPI (blue). The image was acquired through fluorescence microscopy at 40× magnification. The dotted line marks the junction of the epidermis (top) and dermis (bottom), and arrows point to intradermal basophils. Basophils in the dermis possess intracellular S1P.

We were able to show that basophils react to S1P in various ways and that the lipid has an effect on the disease pathophysiology of atopy. Therefore, we wanted to investigate if basophils are a source of S1P that could act in autocrine manners. In the immunofluorescence staining of an AD skin biopsy with anti-S1P and the basophil identifying anti-2D7, we could identify two basophils in the dermis of the sample that were S1P positive (Figure 6).

## 3. Discussion

S1P is emerging as an important mediator in immune and inflammatory disorders [56], and the expression of the G-protein coupled S1PR has been studied for various immune cells like mast cells, eosinophils, and neutrophils. The effects of stimulating S1PR-expressing cells with S1P range from chemotaxis in eosinophils and neutrophils [52,53,57] to amplification of degranulation and cytokine release in mast cells [47,58] and the redirection of neutrophil apoptosis to the formation of neutrophil extracellular traps (NETs) during liver injury [59]. As basophils are the only type of granulocyte in which S1PR expression has yet to be studied, we were curious to see if we could produce similar findings to eosinophils and neutrophils in receptor expression and function for basophils.

The results presented in this study, therefore, provide the first data on S1P and basophils in conjunction. While eosinophils have been found to express all five S1PRs [52,60] and neutrophils S1PR1, S1PR3 and S1PR4 [53,57,60], we confirmed the expression of S1PR1, S1PR2, S1PR3 and S1PR4 on the mRNA level of basophils. We were also able to detect the expression of all five receptors in HEK 293 cells, demonstrating the functionality of our experimental set-up. mRNA expression levels of the S1PRs are comparable in non-atopic and atopic patients. Basophils expressing these receptors provide multiple points of interaction and thus the possibility of a wide variety of functions, leading us to perform the experiments in this paper in order to discover the biological effects. As both basophils and S1P are key players in atopic diseases, we also wanted to elucidate possible implications of interaction for these diseases.

As basophil activation often results in degranulation and subsequent cytokine release, and S1P has been found to influence degranulation in mast cells [47,58], we first performed a BAT to investigate whether stimulation with S1P affects this aspect of basophil function. Protein expressions of CD63 and CD203c, which respectively are markers for anaphylactic and piecemeal degranulation [61], were evaluated through flow cytometry analysis with different doses of S1P. In both cases, differences in marker expression could not be detected, indicating that binding of S1P to the cell surface receptors is unlikely to induce IgE-dependant or independent degranulation in basophils. Further, measurement of intracellular calcium levels with a Fluo-4 real-time calcium flux assay revealed no immediate mobilization of Ca^2+^ into the cytoplasm. Pre-incubating the basophils with IL-3 provided similar results. Performing the same Ca^2+^ flux experiment with HEK 293 cells results in a visible change in cytosolic calcium levels, confirming the functionality of this experimental set-up. Together these findings for basophils indicate that biological effects of S1P binding to the S1PRs do not manifest in a short time frame, and longer stimulation times might be needed to investigate and determine S1P-induced effects.

To determine S1P’s effects on cell apoptosis and life span, we performed an annexin V-FITC and PI apoptosis assay after incubating basophils with various doses of S1P. As 4 h of incubation were unsuccessful in inducing measurable apoptotic effects even in the positive control group and depending on the potency of stimuli, different time frames can be necessary to determine effects [62], we extended the incubation period to 24 h. Here we could observe that the percentage of viable basophils was significantly reduced by 10 µM S1P, while the number of combined apoptotic and late apoptotic basophils was increased. The low dose of 0.1 µM S1P affected basophils in an anti-apoptotic manner by markedly decreasing the percentage of combined apoptotic cells after 24 h of incubation. This is congruent with findings in other cell types, such as those of Bonnaud et al. (2010), where 1 µM S1P exhibits anti-apoptotic effects in endothelial cells associated with activation of the AKT pathway [63]. A study in murine fibroblasts conducted by Castillo et al. (2003) also confirmed that 5 µM S1P inhibits apoptosis through ERK activation [64]. The phenomenon of S1P exhibiting the opposite effect on apoptosis and becoming pro-apoptotic with the higher dose of 10 µM might be due to this dose being outside of physiologically typical concentrations and entering the realm of toxicity [48,65]. As S1P levels can be elevated in different tissues involved in atopic diseases, induction of basophil apoptosis might be a protective measure to limit the involvement of these cells and regulate levels of inflammation.

Basophils majorly contribute to the process of allergic inflammation by orchestrating eosinophil chemotaxis to affected areas [3]. Additionally, basophils themselves are also able to migrate in response to chemotactic compounds [66]. This is often mediated through the chemokine receptor 3 (CCR3) in response to the chemo attractants eotaxin and monocyte chemotactic protein 4 (MCP-4) [67] but has also been observed to be inducible through activation of the histamine H4 receptor [68]. Roviezzo et al. (2004) demonstrated that S1PR signaling influences chemotaxis in eosinophils [52], while Rahaman et al. (2006) showed the same for neutrophil granulocytes [53]. We thus thought it likely that the S1P/S1PR signaling axis could initiate similar responses in basophils. Therefore, we utilized a transwell migration assay to evaluate basophil migration in response to different doses of S1P. While basophils from non-atopic donors seem to migrate slightly towards S1P in a dose dependent manner, though this is only a trend and not statistically significant, chemotaxis is clearly inhibited in basophils from atopic patients. As many IgE-sensitized individuals exhibit no typical symptoms of allergic diseases [69,70], and the prevalence of allergic sensitization is high in the western world [71], underlying unknown allergies might be present in some patients of the non-atopic group. We, therefore, cannot confidently determine if the measured parameters accurately represent the behavior of cells in truly healthy patients.

The fugetactic effect in basophils from atopic patients might well be due to the reduced expression of S1PR1 as this receptor is heavily involved in cell migration of various immune cells such as mast cells [55], eosinophils [72], and neutrophils [73]. The reduction in receptor expression might provide interesting insights into disease pathophysiology. As the difference in S1PR1 expression is observable on the protein but not mRNA level, it is likely due to post-transcriptional regulatory processes [74]. The S1P-induced reduced numbers of migrated basophils, which mainly exhibit pro-inflammatory effects, imply an anti-inflammatory effect of S1P. Therefore, the increased levels in BALF of asthmatic patients [44], and increased serum levels in AD patients [50], might prevent the exacerbation of inflammatory symptoms by reducing basophil involvement. Basophils also contain S1P in their granules, and the resulting possibility of autocrine signaling might be a further indication of S1P limiting the inflammatory response of basophils. As AD is associated with increased basophil infiltration into the skin [75,76], the release of intracellular S1P might have an effect on controlling basophil numbers in the surrounding tissue.

In conclusion, we were able to detect the expression of four S1P receptors in basophils, of which the expression of S1PR1 is reduced in atopic patients. Further, we were able to demonstrate the anti-apoptotic effects of S1P on basophils in low doses and apoptotic effects in high doses. Additionally, basophil migration in response to S1P is reduced in atopic patients, and basophils in AD skin possess intracellular S1P. Together these results imply that the interaction of S1P and basophils might limit inflammation in atopic diseases, providing important data that might aid in understanding pathological processes in these conditions. Further research is needed to fully understand the effect of S1P on basophils and the implications for atopic conditions. Future experiments, such as performing cytokine release assays, might help elucidate the relevance of S1P’s interaction with basophils in atopic diseases.

## 4. Materials and Methods

### 4.1. Collection of Blood Samples

Blood was collected from consenting patients at the Klinikum Oldenburg AöR (approved by the local medical ethics committee, ref.# 2017-109 and ref.# 2021-025) by qualified health professionals. Patients were categorized either into the non-atopic or atopic group. To qualify as “healthy,” patients were required to be non-atopic and free of any chronic inflammatory diseases. Patients with type I hypersensitivities, such as allergic rhinitis and atopic dermatitis, were included in the “atopic” group. All patients were required to have received no treatments, such as antihistamines or corticosteroids, in the two weeks prior to blood collection. Non-atopic patients had a mean age of 43.5 years (SEM = 3.62; *n* = 27, male = 11, female = 16), while the atopic group had an average age of 40.6 years (SEM = 4.29; *n* = 11, male = 4, female = 7).

### 4.2. Basophil Isolation

Basophils were isolated from whole blood collected in EDTA tubes using the EasySep™ direct human basophil isolation kit (STEMCELL Technologies, Vancouver, BC, Canada). The amounts of both the isolation cocktail and rapid spheres recommended by the manufacturer were halved, but an additional fourth isolation step was added to increase basophil purity. Cell counts and purities were determined by staining the cells with anti-CD123 pacific blue (PB) (RRID: AB_2750165) and anti-FcεRIα allophcocyanin (APC) (RRID: AB_10578086) specific flow cytometry antibodies (BioLegend, San Diego, CA, USA), and analyzing the parameters with the CytoFlex S system and Kaluza analysis software version 2.1.1 (Beckmann Coulter, Brea, CA, USA), which were used for all flow cytometry analyses mentioned in this paper. Basophil purity was consistently measured to be ≥96%, and viability determined through 7-AAD (130-111-568) (Miltenyi Biotec, Bergisch Gladbach, Germany) staining always exceeded 99%.

### 4.3. Quantitative Real-Time PCRs

RNA was isolated from ≥96% pure basophils using the High Pure RNA Isolation Kit (Roche, Basel, Switzerland) according to the manufacturer’s instructions and eluted in 50 µL of elution buffer. Subsequently, cDNA was synthesized with the Transcriptor First Strand cDNA Synthesis Kit (Roche, Basel, Switzerland) following the recommended protocol and using the random hexamer primer. mRNA gene expression was analyzed through quantitative real-time PCR using the LightCycler 96 system (Roche, Basel, Switzerland), TaqMan FAM-MGB probes (human glyceraldehyde-3-phosphate dehydrogenase (GAPDH) (Hs00924881_m1), human S1PR1 (Hs00173499_m1); human S1PR2 (custom; forward: 5′-CGTCTTTATCGTCTGCTGGCT-3′, reverse: 5′-GGACAGGCATAGTCCAGAAGGA-3′, probe: CCGCCTTCAGCATCC); human S1PR3 (Hs01019574_m1); human S1PR4 (Hs02330084_s1); human S1PR5 (Hs00924881_m1) (ThermoFisher Scientific, Waltham, MA, USA) and the FastStart TaqMan Probe Master (Roche, Basel, Switzerland). For all analyses, samples were preincubated at 95 °C for 600 s and subsequently amplified through a 2-step protocol, consisting of 10 s at 95 °C followed by 30 s at 60 °C. Gene expressions were normalized to the housekeeping gene GAPDH. 

### 4.4. Basophil Activation Test (BAT)

In order to determine basophil activation, basophils were resuspended in RPMI medium (containing 10% FCS and 1% PenStrep), to which the stimuli diluted in RPMI were added. The concentration of S1P stemming from the FCS in the RPMI medium is approximately 35 nM [77]. As this constitutes less than half of the lowest S1P concentration applied to basophils, it is unlikely to affect results. For d18:1 S1P (860492P) (Avanti Polar Lipids, Alabaster, AL, USA), final concentrations of 0.1, 1, and 10 µM dissolved in a 4 mg/mL fatty acid free BSA solution were used. The positive control consisted of 1 µg/mL a-IgE (16284) (Sigma-Aldrich, St. Louis, MO, USA) and the same amount of fatty acid free BSA in RPMI as the negative control. Cells were then incubated at 37 °C and 5% CO_2_ for 30 min and thereafter placed on ice. After centrifugation, supernatants were removed and cells stained with anti-CD63 FITC (RRID: AB_10898319) and anti-CD203c PE (AB_756044) antibodies (BioLegend, San Diego, CA, USA) in addition to the basophil identifying anti-CD123 PB and anti-FcεRIα APC antibodies for 10 min. The percentages of marker-positive cells were measured by flow cytometry. Isotype and fluorescence minus one (FMO) controls were used for each antibody, and gates were set accordingly.

### 4.5. Calcium-Flux Experiments

Experiments to detect changes in calcium flux were performed using the CytoFlex S platform. For this, after isolation, ≥96% pure cells were resuspended in RPMI medium, loaded with 4 µM Fluo-4, AM (F14217) (ThermoFisher Scientific, Waltham, MA, USA), and incubated at 37 °C and 5% CO_2_ for 20 min. Subsequently, cells were centrifuged, and the supernatants containing excess dye were removed. Pellets were then resuspended in RPMI without phenol red, and if preincubation with IL-3 (200-3) (PeproTech, Cranbury, NJ, USA) occurred, 10 ng/mL IL-3 added and incubated at 37 °C and 5% CO_2_ for a further 20 min. Cells were then placed in the flow cytometry machine, the measurement commenced, and left to run for 1 min to establish a stable baseline. Subsequently, stimuli were added to the running measurement, and changes in fluorescence induced through differential intracellular Ca^2+^ levels measured for 300 s. 500 nM ionomycin (I24222) (ThermoFisher Scientific, Waltham, MA, USA) was used as the positive and the fatty acid free BSA solution used for the S1P, utilized as the negative control. S1P was applied in concentrations of 0.1, 1, and 10 µM. Data were calculated by subtracting the baseline value from the values obtained after the application of stimuli.

### 4.6. Apoptosis Assay

Isolated basophils with a purity of ≥ 98% were resuspended in RPMI medium and stimulated with 0.1, 1, or 10 µM S1P, 1 µM staurosporine (J62837.MCR) (ThermoFisher Scientific, Waltham, MA, USA), or RPMI with fatty acid-free BSA for 24 h at 37 °C and 5% CO_2_. Basophils were then placed on ice, and an annexin V-FITC and propidium iodide (PI) kit (IM2375) (Immunotech SAS, Marseille, France) was used according to the manufacturer’s instructions to stain phosphatidylserine and DNA and determine the stage of apoptosis (viable cells, early apoptosis, late apoptosis, necrosis). Percentages of cells in the different stages of apoptosis were detected using flow cytometry analysis.

### 4.7. Chemotaxis Assay

Basophils were resuspended in RPMI with FCS and placed in the upper chamber of a 24-well plate, 8 µm, PC transwell insert (782717) (BrandTech Scientific, Essex, CT, USA). Stimuli diluted in RPMI without FCS were added to the bottom part of the 24-well plate immediately after, with 8 ng/mL eotaxin (300-21) (PeproTech, Cranbury, NJ, USA) used as the positive control and RPMI with fatty acid free BSA solution utilized as the negative control. S1P concentrations were either 1 or 10 µM. Following 3 h at 37 °C and 5% CO_2_ of incubation, transmigrated cells in the bottom chamber were collected, and the number of cells was determined through staining with the basophil identifying anti-CD123 PB and anti-FcεRIα APC antibodies and flow cytometry analysis. Transmigration rates were calculated in relation to the negative control. A chemotactic index (CI) of >1 indicates the chemotactic activity of a stimulus, while an index of <1 suggests anti-chemotactic properties.

### 4.8. S1PR1 Protein Expression Analysis

S1PR1 protein expression on basophils was determined through staining cells with an anti-S1PR1 PE (NB110-93513PE) (Novus Biologicals, Littleton, CO, USA) specific antibody, in addition to the basophil identifying anti-CD123 PB and anti-FcεRIα APC antibodies for 10 min, and subsequently measuring the corresponding fluorescence through flow cytometry. The unspecific binding of antibodies was determined using respective isotype controls and gates for identifying the percentages of positive basophil populations set accordingly. Background fluorescence was quantified through the use of fluorescence minus one (FMO) controls.

### 4.9. Immunofluorescence Staining of AD Skin Sections

Lesional AD skin biopsies were cryo-sectioned to 6 µm thickness, fixed with 4% PFA, and blocked with a 2x Casein solution (B6429) (Sigma-Aldrich, St. Louis, MO, USA). Slides were consecutively incubated anti-S1P (1:200) (Z-P300) (Echelon Biosciences, Salt Lake City, UT, USA), goat anti-mouse Alexa Fluor 488 secondary antibody (1:2000) (15607878) (ThermoFisher Scientific, Waltham, MA, USA) and the basophil specific anti-2D7 Alexa Fluor 647 (RRID: AB_1967142) (1:100) (BioLegend, San Diego, CA, USA). Slides stained with the respective isotypes or only the secondary antibody were utilized as experimental controls. Slides were subsequently mounted with Fluoromount-G Mounting Medium with DAPI (00-4959-52) (ThermoFisher Scientific, Waltham, MA, USA) and analyzed with the Olympus BX63 fluorescence microscope and the CellSens image analysis software (Olympus, Tokyo, Japan).

### 4.10. Statistical Analysis

Data were statistically analyzed using version 9.2.0 of the statistics software Prism (GraphPad, San Diego, CA, USA). All values are depicted as mean ± standard error of the mean (SEM). One-way ANOVAs followed by a Dunnet post hoc test or paired and unpaired t-tests (two-tailed) were used to calculate the significances between groups. *p*-values of <0.05 were considered to be statistically significant (* ≤ 0.05; ** ≤ 0.01; *** ≤ 0.001).

## Data Availability

Not applicable.

## Abbreviations

APC	allophycocyanin	
AD	atopic dermatitis	
a-IgE	anti-IgE	
AT	atopic	
BALF	bronchoalveolar lavage fluid	
BAT	basophil activation test	
BSA	bovine serum albumin	
Ca	calcium	
CCR3	chemokine receptor 3	
CI	chemotactic index	
CO_2_	carbon dioxide	
cDNA	complementary deoxyribonucleic acid	
EDTA	ethylenediaminetetraacetic acid	
FCS	fetal calf serum	
FMO	fluorescence minus one	
GAPDH	glyceraldehyde-3-phosphate dehydrogenase	
h	hours	
IgE	immunoglobulin E	
IL-3	interleukin 3	
IL-4	interleukin 4	
IL-10	interleukin 10	
IL-13	interleukin 13	
IL-31	interleukin 31	
LTC4	leukotriene 4	
MCP-4	monocyte chemotactic protein 4	
MFI	mean fluorescence intensity	
min	minutes	
mRNA	messenger ribonucleic acid	
NA	non-atopic	
NET	neutrophil extracellular traps	
NK	natural killer	
PB	pacific blue	
PC	polycarbonate	
PE	phycoerythrin	
PI	propidium iodide	
RNA	ribonucleic acid	
s	seconds	
S1P	sphingosine-1-phosphate	
S1PR	sphingosine-1-phosphate receptor	
SEM	standard error of the mean	
TALL	T cell acute lymphoblastic leukemia	
T_h_2	T helper cell type 2	
TSLP	thymic stromal lymphopoietin	
qRT-PCR	quantitative real-time polymerase chain reaction	
